# Di-μ_2_-acetato-1:2κ^2^ *O*:*O*′;2:3κ^2^ *O*:*O*′-bis­(*N*,*N*′-dimethyl­formamide)-1κ*O*,3κ*O*-bis­{μ_2_-2,2′-[propane-1,3-diylbis(imino­methyl­ene)]diphenolato-1κ^4^ *O*,*N*,*N*′,*O*′:2κ^2^ *O*,*O*′;2κ^2^ *O*,*O*′:3κ^4^ *O*,*N*,*N*′,*O*′-1,3-dinickel(II)-2-cadmium(II)

**DOI:** 10.1107/S1600536807067724

**Published:** 2008-01-04

**Authors:** Leyla Tatar Yıldırım, Orhan Atakol

**Affiliations:** a Department of Physics Engineering, Hacettepe University, 06800 Beytepe, Ankara, Turkey; b Department of Chemistry, Ankara University, Ankara, Turkey

## Abstract

The crystal structure of the title compound, [Ni_2_Cd(C_17_H_16_N_2_O_2_)_2_(C_2_H_3_O_2_)_2_(C_3_H_7_NO)_2_], contains discrete centrosymmetric hetero-trinuclear mol­ecules in which Ni/Cd atom pairs are triply bridged *via* O atoms from the SALPD^2−^ [*N*,*N*′-bis­(salicyl­idene)-1,3-propane­diaminate] and acetate ligands. The central Cd^II^ ion is in a distorted octa­hedral coordination environment formed by four O atoms from two SALPD^2−^ ligands in the equatorial plane and two O atoms of two symmetry-related acetate ligands in the axial positions. The symmetry-related Ni^II^ ions are in slightly distorted octa­hedral environments, coordinated by two O and two N atoms from tetra­dendate SALPD^2−^ ligands in the equatorial plane, while the axial positions are occupied by O atoms from a dimethyl­formamide and an acetate ligand. This results in the formation of three edge-shared octa­hedra in which the Ni⋯Cd distance is 3.1482 (15) Å. The crystal structure is stabilized by weak C—H⋯O hydrogen bonds.

## Related literature

For general background, see: Aneetha *et al.* (1999); Reglinski *et al.* (2006 ▶); Fukuhara *et al.* (1990 ▶); Barandika *et al.* (1999 ▶). For related literature, see: Aneetha *et al.* (1999 ▶); Atakol *et al.* (1999 ▶);Ülkü *et al.* (1999 ▶); Ülkü *et al.* (2001 ▶); Tatar & Atakol (2002 ▶); Tatar Yıldırım *et al.* (2007 ▶); Tatar Yıldırım & Ergün (2007 ▶).
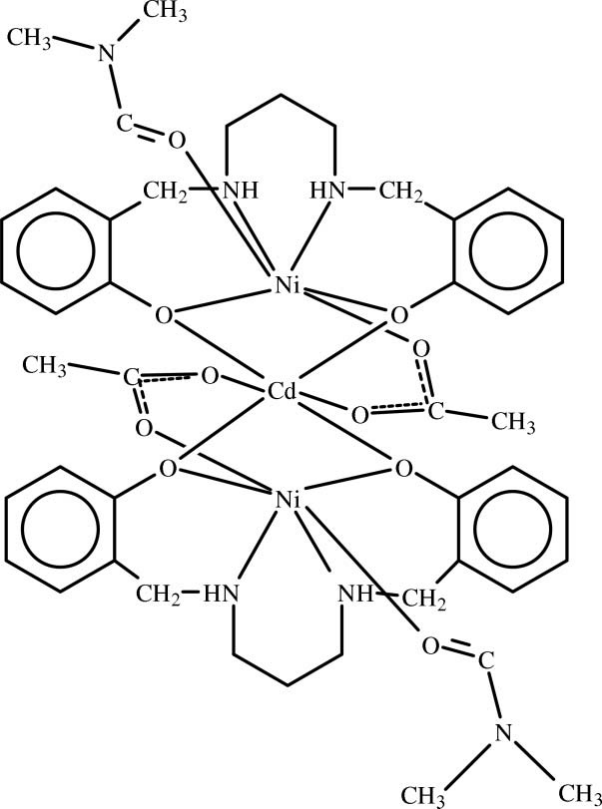


## Experimental

### 

#### Crystal data

[Ni_2_Cd(C_17_H_16_N_2_O_2_)_2_(C_2_H_3_O_2_)_2_(C_3_H_7_NO)_2_]
*M* *_r_* = 1062.77
Monoclinic, 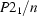

*a* = 10.285 (3) Å
*b* = 18.040 (5) Å
*c* = 12.590 (3) Å
β = 92.12 (2)°
*V* = 2334.4 (11) Å^3^
*Z* = 2
Mo *K*α radiation
μ = 1.31 mm^−1^
*T* = 295 (2) K
0.4 × 0.1 × 0.1 mm

#### Data collection

Enraf–Nonius TurboCAD-4 diffractometer
Absorption correction: ψ scan (North *et al.*, 1968 ▶) *T*_min_ = 0.854, *T*_max_ = 0.877
3960 measured reflections
3612 independent reflections
1997 reflections with *I* > 2σ(*I*)
*R*_int_ = 0.076
3 standard reflections frequency: 120 min intensity decay: 1%

#### Refinement

*R*[*F* ^2^ > 2σ(*F* ^2^)] = 0.071
*wR*(*F* ^2^) = 0.239
*S* = 1.02
3612 reflections
287 parameters
H-atom parameters not refined
Δρ_max_ = 0.88 e Å^−3^
Δρ_min_ = −1.51 e Å^−3^

### 

Data collection: *CAD-4 EXPRESS* (Enraf–Nonius, 1994 ▶); cell refinement: *CAD-4 EXPRESS*; data reduction: *XCAD4* (Harms & Wocadlo, 1995 ▶); program(s) used to solve structure: *SHELXS97* (Sheldrick, 1997 ▶); program(s) used to refine structure: *SHELXL97* (Sheldrick, 1997 ▶); molecular graphics: *ORTEP-3 for Windows* (Farrugia, 1997 ▶); software used to prepare material for publication: *WinGX* (Farrugia, 1999 ▶).

## Supplementary Material

Crystal structure: contains datablocks I, global. DOI: 10.1107/S1600536807067724/lh2581sup1.cif

Structure factors: contains datablocks I. DOI: 10.1107/S1600536807067724/lh2581Isup2.hkl

Additional supplementary materials:  crystallographic information; 3D view; checkCIF report

## Figures and Tables

**Table 1:** Selected bond lengths (Å)

Cd—O1	2.157 (7)
Cd—O2	2.151 (7)
Cd—O3	2.212 (7)
Ni—N1	2.094 (9)
Ni—N2	2.101 (9)
Ni—O1	2.031 (8)
Ni—O2	2.060 (7)
Ni—O4	2.045 (7)
Ni—O5	2.180 (7)

**Table 2:** Hydrogen-bond geometry (Å, °)

*D*—H⋯*A*	*D*—H	H⋯*A*	*D*⋯*A*	*D*—H⋯*A*
C2—H2⋯O3^i^	0.93	2.56	3.308 (16)	138
C20—H20⋯O3^i^	0.93	2.59	3.409 (14)	147

Symmetry code: (i) 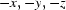.

**Fig. 1.**
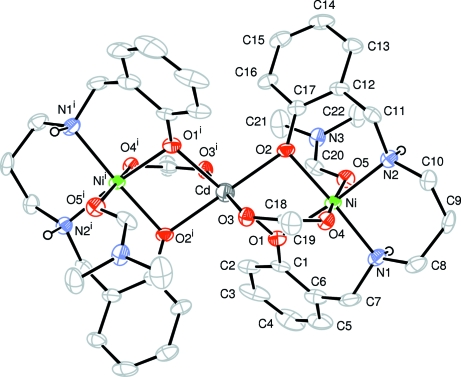
The molecular structure of the title molecule with the atom-numbering scheme. Displacement ellipsoids are drawn at the 30% probability level (symmetry code (i): -x, -y, -z). H atoms bonded to C atoms are not shown.

## supplementary crystallographic information

### Comment

Recently, ONNO phenol amines have been reported by the reduction of ONNO type Schiff bases (Aneetha *et al.*, 1999, Reglinski *et al.*, 2006). Bis-*N*,*N*'(2-salicylidene)-1,3-propanediamine is a ligand which tends to give polynuclear complexes. Reduction of this Schiff base results in the formation of bis-*N*,*N*'(2-hydroxybenzyl)-1, 3-propanediamine. The ONNO type ligand stereochemistry around metal ions and the structure of the O-atom bridges influence the magnetic exchange interactions (Barandika *et al.*, 1999; Fukuhara *et al.*, 1990).

This study deals with the investigation of the production of hetero-trinuclear complexes with the use of bis-*N*,*N*'(2-hydroxybenzyl)-1,3-propanediamine and it was observed that Ni(II)—Cd(II)—Ni(II) type complexes were formed. The crystal structure of the title compound (I), contains a linear Ni—Cd—Ni trinuclear complex with a central Cd^II^ ion located on an inversion centre and terminal Ni^II^ ions related by this inversion centre (Fig. 1). The Cd^II^ ion has a distorted octahedral coordination environment, formed by four O atoms from two SALPD^2-^ ligands in the equatorial plane and two O atoms from two acetate ligands at the axial positions. The coordination bond lengths and angles around the Cd^II^ ion range between 2.151 (7) - 2.212 (7)Å and 78.4 (3) - 101.6 (3)°, respectively.

The terminal Ni^II^ ions have slightly distorted octahedral coordination environments formed by two O atoms and two N atoms from SALPD^2-^ ligands in the equatorial plane and two O atoms from acetate ligand and dimethylformamide ligand at the axial positions. In the Ni coordination sphere bond lengths and angles range between 2.031 (8) - 2.180 (7)Å and 83.3 (3) - 93.8 (3)°, respectively.

The overall result is three edge-shared octahedral in which the closest Ni···Cd distance is 3.1482 (15) Å. The crystal structure is stabilized by weak C—H···O hydrogen bonds.

The coordination geometry of the metal ions is very similar to those found for the corresponding complexes reported previously (Tatar & Ergün, 2007; Tatar & Atakol *et al.*, 2007; Tatar & Atakol 2002; Atakol *et al.*, 1999;Ülkü *et al.*, 1999;Ülkü *et al.*, 2001).

### Experimental

The related Schiff Base, bis-*N*,*N*'(salicylidene)-1,3-propanedimine was prepared through the condensation reaction of an 1,3-propanediamine and salicylaldehyde in EtOH and this Schiff bases prepared was reduced with NaBH_4_ in MeOH until the solution was completely colorless. The Phenolic amine ligand was precipitated with the addition of excess ice.

The complex was prepared with template method since it was very cumbersome to isolate mononuclear bis-*N*,*N*'(2-oxybenzyl)-1,3-propanediaminato nickel(II) complex. 0.568 g (0.002 mole) bis-*N*,*N*'(2-hydroxybenzyl)-1,3-propanediamine was dissolved in 50 ml hot DMF. 0.475 g(0.002 mole) NiCl_2_.6H_2_O solution in 20 ml hot methanol and 0.5 ml Et_3_N were added to it and the mixture was stirred for ten minutes. Then a solution of 0.312 g (0.001 mol) Cd(CH_3_COO)_2_.4H_2_O in 20 ml hot methanol was added and the resulting mixture was kept on the bench for 3–4 days. The blue crystals were filtered off and dried in air.

### Refinement

H atoms were positioned geometrically, with C—H = 0.93, 0.97 and 0.96 Å for aromatic, methylene and methyl H, and constrained to ride on their parent atoms, with *U*_iso_(H) = *xU*_eq_(C), where *x* = 1.5 for methyl H, and *x* = 1.2 for all other H atoms. The fact that only *ca* 87% of the available data were collected to a maximun 2θ of 50° can lower the precision of the structure.

### Crystal data

**Table:**

[Ni_2_Cd(C_17_H_16_N_2_O_2_)_2_(C_2_H_3_O_2_)_2_(C_3_H_7_NO)_2_]	*F*_000_ = 1100
*M**_r_* = 1062.77	*D*_x_ = 1.512 Mg m^−^^3^
Monoclinic, *P*2_1_/*n*	Mo *K*α radiation λ = 0.71073 Å
Hall symbol: -P 2yn	Cell parameters from 15 reflections
*a* = 10.285 (3) Å	θ = 10.0–11.1º
*b* = 18.040 (5) Å	µ = 1.31 mm^−^^1^
*c* = 12.590 (3) Å	*T* = 295 (2) K
β = 92.12 (2)º	Prism, blue
*V* = 2334.4 (11) Å^3^	0.4 × 0.1 × 0.1 mm
*Z* = 2	

### Data collection

**Table:**

Enraf–Nonius TurboCAD-4 diffractometer	θ_max_ = 25.1º
non–profiled ω scans	θ_min_ = 2.5º
Absorption correction: ψ scan(North *et al.*, 1968)	*h* = 0→12
*T*_min_ = 0.854, *T*_max_ = 0.877	*k* = 0→21
3960 measured reflections	*l* = −14→12
3612 independent reflections	3 standard reflections
1997 reflections with *I* > 2σ(*I*)	every 120 min
*R*_int_ = 0.076	intensity decay: 1%

### Refinement

**Table:**

Refinement on *F*^2^	Secondary atom site location: difference Fourier map
Least-squares matrix: full	Hydrogen site location: geomtr
*R*[*F*^2^ > 2σ(*F*^2^)] = 0.072	H-atom parameters not refined
*wR*(*F*^2^) = 0.239	*w* = 1/[σ^2^(*F*_o_^2^) + (0.1184*P*)^2^ + 12.1765*P*] where *P* = (*F*_o_^2^ + 2*F*_c_^2^)/3
*S* = 1.02	(Δ/σ)_max_ < 0.001
3612 reflections	Δρ_max_ = 0.88 e Å^−^^3^
287 parameters	Δρ_min_ = −1.51 e Å^−^^3^
Primary atom site location: structure-invariant direct methods	Extinction correction: none

### Special details

**Table:**

Geometry. All e.s.d.'s (except the e.s.d. in the dihedral angle between two l.s. planes) are estimated using the full covariance matrix. The cell e.s.d.'s are taken into account individually in the estimation of e.s.d.'s in distances, angles and torsion angles; correlations between e.s.d.'s in cell parameters are only used when they are defined by crystal symmetry. An approximate (isotropic) treatment of cell e.s.d.'s is used for estimating e.s.d.'s involving l.s. planes.
Refinement. Refinement of *F*^2^ against ALL reflections. The weighted *R*-factor *wR* and goodness of fit *S* are based on *F*^2^, conventional *R*-factors *R* are based on *F*, with *F* set to zero for negative *F*^2^. The threshold expression of *F*^2^ > σ(*F*^2^) is used only for calculating *R*-factors(gt) *etc*. and is not relevant to the choice of reflections for refinement. *R*-factors based on *F*^2^ are statistically about twice as large as those based on *F*, and *R*- factors based on ALL data will be even larger.

### Fractional atomic coordinates and isotropic or equivalent isotropic displacement parameters (Å^2^)

**Table:**

	*x*	*y*	*z*	*U*_iso_*/*U*_eq_	
Cd	0	0	0	0.0489 (4)	
Ni	0.03480 (12)	0.14386 (8)	−0.13762 (11)	0.0301 (4)	
O5	−0.1184 (7)	0.1304 (4)	−0.2605 (6)	0.0409 (19)	
O2	−0.0909 (6)	0.1061 (4)	−0.0260 (6)	0.0368 (18)	
O1	0.0874 (7)	0.0355 (4)	−0.1446 (6)	0.0383 (18)	
N2	−0.0396 (9)	0.2521 (5)	−0.1294 (7)	0.038 (2)	
H2N	−0.0967	0.2576	−0.1858	0.045*	
N1	0.1589 (9)	0.1727 (6)	−0.2591 (8)	0.044 (3)	
H1N	0.1088	0.1762	−0.32	0.053*	
C12	−0.2316 (10)	0.2091 (7)	−0.0309 (9)	0.041 (3)	
C11	−0.1159 (10)	0.2610 (6)	−0.0316 (10)	0.041 (3)	
H11A	−0.1463	0.3118	−0.0272	0.05*	
H11B	−0.0598	0.2514	0.0304	0.05*	
C1	0.1117 (10)	0.0039 (7)	−0.2371 (10)	0.041 (3)	
C17	−0.2108 (10)	0.1325 (6)	−0.0246 (8)	0.035 (3)	
N3	−0.3098 (9)	0.0735 (5)	−0.2942 (8)	0.044 (3)	
C16	−0.3197 (10)	0.0877 (7)	−0.0137 (10)	0.044 (3)	
H16	−0.3081	0.037	−0.0039	0.053*	
C8	0.2249 (11)	0.2441 (8)	−0.2442 (11)	0.055 (4)	
H8A	0.2818	0.2522	−0.3028	0.065*	
H8B	0.2786	0.2422	−0.1793	0.065*	
C6	0.1915 (11)	0.0396 (8)	−0.3095 (10)	0.053 (3)	
C20	−0.1877 (10)	0.0758 (7)	−0.2586 (9)	0.043 (3)	
H20	−0.1519	0.0326	−0.2298	0.051*	
C10	0.0577 (11)	0.3126 (6)	−0.1368 (10)	0.047 (3)	
H10A	0.1193	0.3093	−0.0768	0.056*	
H10B	0.0136	0.36	−0.1326	0.056*	
C5	0.2079 (15)	0.0093 (10)	−0.4079 (12)	0.077 (5)	
H5	0.2562	0.0355	−0.4563	0.092*	
C2	0.0636 (12)	−0.0651 (8)	−0.2658 (11)	0.059 (4)	
H2	0.0165	−0.092	−0.2172	0.071*	
C9	0.1305 (11)	0.3096 (7)	−0.2378 (11)	0.051 (4)	
H9A	0.1791	0.3553	−0.2448	0.061*	
H9B	0.0681	0.3068	−0.2974	0.061*	
C15	−0.4443 (11)	0.1161 (8)	−0.0171 (10)	0.054 (3)	
H15	−0.5152	0.0844	−0.0121	0.064*	
C21	−0.3905 (14)	0.0079 (8)	−0.2850 (14)	0.085 (6)	
H21A	−0.4761	0.018	−0.3146	0.128*	
H21B	−0.3963	−0.0052	−0.2114	0.128*	
H21C	−0.3526	−0.0324	−0.3228	0.128*	
C22	−0.3729 (13)	0.1390 (8)	−0.3350 (13)	0.072 (5)	
H22A	−0.4613	0.1276	−0.3561	0.108*	
H22B	−0.3278	0.1568	−0.3953	0.108*	
H22C	−0.372	0.1765	−0.2808	0.108*	
C13	−0.3563 (11)	0.2387 (8)	−0.0343 (9)	0.049 (3)	
H13	−0.369	0.2896	−0.0408	0.058*	
O4	0.1691 (7)	0.1684 (4)	−0.0189 (6)	0.0365 (18)	
O3	0.1560 (7)	0.0659 (4)	0.0804 (6)	0.0378 (18)	
C18	0.1911 (9)	0.1297 (7)	0.0620 (9)	0.035 (3)	
C14	−0.4637 (12)	0.1902 (9)	−0.0278 (10)	0.061 (4)	
H14	−0.5479	0.2091	−0.0308	0.074*	
C3	0.0837 (15)	−0.0952 (10)	−0.3644 (15)	0.083 (6)	
H3	0.0483	−0.1412	−0.3821	0.1*	
C4	0.157 (2)	−0.0572 (12)	−0.4381 (14)	0.093 (6)	
H4	0.1702	−0.0767	−0.5052	0.112*	
C7	0.2531 (12)	0.1124 (8)	−0.2753 (11)	0.060 (4)	
H7A	0.3033	0.1046	−0.2095	0.071*	
H7B	0.3129	0.1279	−0.3288	0.071*	
C19	0.2689 (12)	0.1683 (7)	0.1523 (10)	0.053 (3)	
H19A	0.2146	0.1748	0.212	0.079*	
H19B	0.298	0.2158	0.1283	0.079*	
H19C	0.3428	0.1384	0.173	0.079*	

### Atomic displacement parameters (Å^2^)

**Table:**

	*U*^11^	*U*^22^	*U*^33^	*U*^12^	*U*^13^	*U*^23^
Cd	0.0419 (7)	0.0521 (8)	0.0523 (10)	−0.0009 (6)	−0.0042 (6)	0.0037 (7)
Ni	0.0200 (6)	0.0433 (8)	0.0269 (8)	−0.0003 (6)	−0.0022 (5)	0.0011 (6)
O5	0.034 (4)	0.050 (5)	0.037 (5)	−0.005 (4)	−0.010 (3)	−0.002 (4)
O2	0.022 (4)	0.047 (5)	0.042 (5)	0.001 (3)	0.002 (3)	0.001 (4)
O1	0.033 (4)	0.044 (5)	0.038 (5)	0.000 (3)	0.002 (3)	−0.005 (4)
N2	0.039 (5)	0.046 (6)	0.029 (6)	−0.002 (4)	−0.003 (4)	0.002 (4)
N1	0.036 (5)	0.060 (7)	0.037 (7)	−0.004 (5)	0.003 (4)	0.010 (5)
C12	0.030 (6)	0.057 (8)	0.036 (8)	0.010 (6)	0.003 (5)	−0.002 (6)
C11	0.036 (6)	0.043 (7)	0.044 (8)	0.013 (5)	−0.007 (5)	0.001 (6)
C1	0.025 (5)	0.052 (7)	0.044 (8)	0.014 (6)	0.001 (5)	−0.009 (6)
C17	0.035 (6)	0.050 (7)	0.020 (6)	0.001 (5)	−0.003 (4)	−0.006 (5)
N3	0.037 (5)	0.053 (6)	0.043 (7)	−0.011 (5)	−0.015 (4)	0.011 (5)
C16	0.028 (6)	0.057 (8)	0.048 (8)	−0.013 (5)	0.005 (5)	−0.008 (6)
C8	0.030 (7)	0.085 (10)	0.048 (9)	−0.004 (7)	0.002 (6)	0.012 (7)
C6	0.037 (7)	0.077 (10)	0.043 (9)	0.016 (7)	−0.003 (6)	−0.011 (7)
C20	0.033 (6)	0.052 (8)	0.040 (8)	0.014 (6)	−0.023 (5)	−0.007 (6)
C10	0.052 (8)	0.041 (7)	0.046 (9)	−0.007 (6)	−0.003 (6)	0.004 (6)
C5	0.080 (11)	0.111 (15)	0.042 (10)	0.042 (10)	0.025 (8)	−0.008 (9)
C2	0.046 (7)	0.070 (10)	0.061 (10)	0.016 (7)	0.014 (6)	−0.013 (8)
C9	0.041 (7)	0.049 (8)	0.061 (9)	−0.023 (6)	−0.017 (6)	0.029 (7)
C15	0.030 (6)	0.082 (10)	0.050 (9)	−0.011 (7)	0.003 (6)	−0.001 (7)
C21	0.074 (10)	0.069 (10)	0.110 (15)	−0.031 (8)	−0.045 (10)	0.018 (9)
C22	0.046 (8)	0.065 (10)	0.103 (13)	−0.009 (7)	−0.028 (8)	0.036 (9)
C13	0.039 (7)	0.070 (9)	0.037 (8)	0.019 (6)	−0.001 (5)	0.005 (6)
O4	0.033 (4)	0.049 (5)	0.028 (5)	−0.005 (3)	−0.010 (3)	0.007 (4)
O3	0.039 (4)	0.041 (5)	0.033 (5)	−0.001 (4)	−0.007 (3)	0.006 (3)
C18	0.020 (5)	0.054 (8)	0.031 (8)	0.009 (5)	−0.006 (4)	−0.010 (6)
C14	0.028 (7)	0.113 (14)	0.043 (9)	0.017 (8)	−0.003 (5)	0.000 (8)
C3	0.055 (10)	0.109 (14)	0.084 (14)	0.037 (9)	−0.017 (9)	−0.055 (11)
C4	0.111 (15)	0.121 (17)	0.048 (12)	0.057 (13)	−0.005 (10)	−0.040 (11)
C7	0.030 (7)	0.092 (11)	0.058 (10)	0.012 (7)	0.016 (6)	0.013 (8)
C19	0.053 (8)	0.058 (8)	0.044 (9)	−0.004 (6)	−0.018 (6)	0.004 (6)

### Geometric parameters (Å, °)

**Table:**

Cd—Ni	3.1482 (15)	C8—H8A	0.97
Cd—O1	2.157 (7)	C8—H8B	0.97
Cd—O2	2.151 (7)	C6—C5	1.370 (19)
Cd—O3	2.212 (7)	C6—C7	1.514 (19)
Ni—N1	2.094 (9)	C20—H20	0.93
Ni—N2	2.101 (9)	C10—C9	1.500 (17)
Ni—O1	2.031 (8)	C10—H10A	0.97
Ni—O2	2.060 (7)	C10—H10B	0.97
Ni—O4	2.045 (7)	C5—C4	1.36 (2)
Ni—O5	2.180 (7)	C5—H5	0.93
Cd—O2^i^	2.151 (7)	C2—C3	1.38 (2)
Cd—O1^i^	2.157 (7)	C2—H2	0.93
Cd—O3^i^	2.212 (7)	C9—H9A	0.97
Cd—Ni^i^	3.1482 (15)	C9—H9B	0.97
O5—C20	1.217 (13)	C15—C14	1.358 (19)
O2—C17	1.323 (12)	C15—H15	0.93
O1—C1	1.328 (13)	C21—H21A	0.96
N2—C10	1.486 (14)	C21—H21B	0.96
N2—C11	1.493 (14)	C21—H21C	0.96
N2—H2N	0.91	C22—H22A	0.96
N1—C8	1.465 (15)	C22—H22B	0.96
N1—C7	1.474 (15)	C22—H22C	0.96
N1—H1N	0.91	C13—C14	1.415 (18)
C12—C13	1.389 (15)	C13—H13	0.93
C12—C17	1.400 (16)	O4—C18	1.248 (13)
C12—C11	1.514 (16)	O3—C18	1.231 (13)
C11—H11A	0.97	C18—C19	1.532 (15)
C11—H11B	0.97	C14—H14	0.93
C1—C2	1.384 (17)	C3—C4	1.40 (3)
C1—C6	1.405 (17)	C3—H3	0.93
C17—C16	1.392 (14)	C4—H4	0.93
N3—C20	1.319 (13)	C7—H7A	0.97
N3—C22	1.435 (15)	C7—H7B	0.97
N3—C21	1.452 (15)	C19—H19A	0.96
C16—C15	1.379 (16)	C19—H19B	0.96
C16—H16	0.93	C19—H19C	0.96
C8—C9	1.534 (17)		
			
O1—Cd—O2	78.4 (3)	C16—C17—C12	117.2 (10)
O1—Cd—O2^i^	101.6 (3)	C20—N3—C22	120.5 (10)
O1—Cd—O3	84.8 (3)	C20—N3—C21	122.6 (11)
O2—Cd—O3	83.9 (3)	C22—N3—C21	116.6 (10)
O1—Ni—O2	83.5 (3)	C15—C16—C17	122.1 (12)
O1—Ni—O4	93.8 (3)	C15—C16—H16	118.9
O1—Ni—N1	92.1 (3)	C17—C16—H16	118.9
O1—Ni—O5	92.8 (3)	N1—C8—C9	113.2 (9)
O2—Ni—N2	92.0 (3)	N1—C8—H8A	108.9
O2—Ni—O5	89.6 (3)	C9—C8—H8A	108.9
O4—Ni—O2	89.8 (3)	N1—C8—H8B	108.9
O4—Ni—N1	93.8 (3)	C9—C8—H8B	108.9
O4—Ni—N2	90.0 (3)	H8A—C8—H8B	107.8
N1—Ni—N2	92.2 (4)	C5—C6—C1	119.9 (14)
N1—Ni—O5	87.3 (3)	C5—C6—C7	122.7 (14)
N2—Ni—O5	83.3 (3)	C1—C6—C7	117.4 (11)
Ni—O1—Cd	97.4 (3)	O5—C20—N3	124.8 (11)
Ni—O2—Cd	96.8 (3)	O5—C20—H20	117.6
O2—Cd—O2^i^	180.0 (4)	N3—C20—H20	117.6
O1^i^—Cd—O2	101.6 (3)	N2—C10—C9	112.6 (9)
O2^i^—Cd—O1^i^	78.4 (3)	N2—C10—H10A	109.1
O1^i^—Cd—O1	180.0 (5)	C9—C10—H10A	109.1
O2—Cd—O3^i^	96.1 (3)	N2—C10—H10B	109.1
O2^i^—Cd—O3^i^	83.9 (3)	C9—C10—H10B	109.1
O1^i^—Cd—O3^i^	84.8 (3)	H10A—C10—H10B	107.8
O1—Cd—O3^i^	95.2 (3)	C4—C5—C6	123.2 (17)
O2^i^—Cd—O3	96.1 (3)	C4—C5—H5	118.4
O1^i^—Cd—O3	95.2 (3)	C6—C5—H5	118.4
O3^i^—Cd—O3	180.0 (6)	C3—C2—C1	121.7 (15)
O2—Cd—Ni	40.53 (19)	C3—C2—H2	119.2
O2^i^—Cd—Ni	139.47 (19)	C1—C2—H2	119.2
O1^i^—Cd—Ni	140.2 (2)	C10—C9—C8	114.1 (10)
O1—Cd—Ni	39.8 (2)	C10—C9—H9A	108.7
O3^i^—Cd—Ni	106.67 (19)	C8—C9—H9A	108.7
O3—Cd—Ni	73.33 (19)	C10—C9—H9B	108.7
O2—Cd—Ni^i^	139.47 (19)	C8—C9—H9B	108.7
O2^i^—Cd—Ni^i^	40.53 (19)	H9A—C9—H9B	107.6
O1^i^—Cd—Ni^i^	39.8 (2)	C14—C15—C16	120.1 (12)
O1—Cd—Ni^i^	140.2 (2)	C14—C15—H15	120
O3^i^—Cd—Ni^i^	73.33 (19)	C16—C15—H15	120
O3—Cd—Ni^i^	106.67 (19)	N3—C21—H21A	109.5
Ni—Cd—Ni^i^	180	N3—C21—H21B	109.5
O2—Ni—N1	174.5 (4)	H21A—C21—H21B	109.5
O1—Ni—N2	174.1 (3)	N3—C21—H21C	109.5
O4—Ni—O5	173.3 (3)	H21A—C21—H21C	109.5
O1—Ni—Cd	42.8 (2)	H21B—C21—H21C	109.5
O4—Ni—Cd	82.2 (2)	N3—C22—H22A	109.5
O2—Ni—Cd	42.7 (2)	N3—C22—H22B	109.5
N1—Ni—Cd	133.7 (3)	H22A—C22—H22B	109.5
N2—Ni—Cd	133.6 (3)	N3—C22—H22C	109.5
O5—Ni—Cd	101.8 (2)	H22A—C22—H22C	109.5
C20—O5—Ni	118.9 (7)	H22B—C22—H22C	109.5
C17—O2—Ni	119.9 (7)	C12—C13—C14	118.8 (13)
C17—O2—Cd	136.0 (7)	C12—C13—H13	120.6
C1—O1—Ni	120.7 (7)	C14—C13—H13	120.6
C1—O1—Cd	135.3 (7)	C18—O4—Ni	125.0 (7)
C10—N2—C11	110.4 (9)	C18—O3—Cd	129.0 (7)
C10—N2—Ni	115.6 (7)	O3—C18—O4	129.0 (10)
C11—N2—Ni	110.1 (7)	O3—C18—C19	115.7 (10)
C10—N2—H2N	106.7	O4—C18—C19	115.2 (10)
C11—N2—H2N	106.7	C15—C14—C13	120.2 (11)
Ni—N2—H2N	106.7	C15—C14—H14	119.9
C8—N1—C7	111.3 (9)	C13—C14—H14	119.9
C8—N1—Ni	114.6 (7)	C2—C3—C4	120.6 (17)
C7—N1—Ni	109.8 (7)	C2—C3—H3	119.7
C8—N1—H1N	106.9	C4—C3—H3	119.7
C7—N1—H1N	106.9	C5—C4—C3	117.3 (15)
Ni—N1—H1N	106.9	C5—C4—H4	121.3
C13—C12—C17	121.3 (11)	C3—C4—H4	121.3
C13—C12—C11	119.2 (11)	N1—C7—C6	114.1 (10)
C17—C12—C11	119.5 (9)	N1—C7—H7A	108.7
N2—C11—C12	112.1 (9)	C6—C7—H7A	108.7
N2—C11—H11A	109.2	N1—C7—H7B	108.7
C12—C11—H11A	109.2	C6—C7—H7B	108.7
N2—C11—H11B	109.2	H7A—C7—H7B	107.6
C12—C11—H11B	109.2	C18—C19—H19A	109.5
H11A—C11—H11B	107.9	C18—C19—H19B	109.5
O1—C1—C2	122.5 (11)	H19A—C19—H19B	109.5
O1—C1—C6	120.4 (11)	C18—C19—H19C	109.5
C2—C1—C6	117.1 (12)	H19A—C19—H19C	109.5
O2—C17—C16	123.1 (10)	H19B—C19—H19C	109.5
O2—C17—C12	119.7 (10)		
			
O2—Cd—Ni—O1	−157.4 (4)	N1—Ni—N2—C10	−41.5 (8)
O2^i^—Cd—Ni—O1	22.6 (4)	O5—Ni—N2—C10	−128.5 (8)
O1^i^—Cd—Ni—O1	180	Cd—Ni—N2—C10	131.5 (7)
O3^i^—Cd—Ni—O1	−77.8 (4)	O4—Ni—N2—C11	−73.7 (7)
O3—Cd—Ni—O1	102.2 (4)	O2—Ni—N2—C11	16.2 (7)
O2—Cd—Ni—O4	98.3 (4)	N1—Ni—N2—C11	−167.5 (7)
O2^i^—Cd—Ni—O4	−81.7 (4)	O5—Ni—N2—C11	105.5 (7)
O1^i^—Cd—Ni—O4	75.7 (4)	Cd—Ni—N2—C11	5.5 (8)
O1—Cd—Ni—O4	−104.3 (4)	O1—Ni—N1—C8	−141.8 (7)
O3^i^—Cd—Ni—O4	177.9 (3)	O4—Ni—N1—C8	−47.9 (8)
O3—Cd—Ni—O4	−2.1 (3)	N2—Ni—N1—C8	42.3 (8)
O2^i^—Cd—Ni—O2	180	O5—Ni—N1—C8	125.5 (8)
O1^i^—Cd—Ni—O2	−22.6 (4)	Cd—Ni—N1—C8	−130.7 (7)
O1—Cd—Ni—O2	157.4 (4)	O1—Ni—N1—C7	−15.7 (8)
O3^i^—Cd—Ni—O2	79.6 (4)	O4—Ni—N1—C7	78.2 (8)
O3—Cd—Ni—O2	−100.4 (4)	N2—Ni—N1—C7	168.4 (8)
O2—Cd—Ni—N1	−173.9 (5)	O5—Ni—N1—C7	−108.4 (8)
O2^i^—Cd—Ni—N1	6.1 (5)	Cd—Ni—N1—C7	−4.6 (10)
O1^i^—Cd—Ni—N1	163.5 (5)	C10—N2—C11—C12	169.7 (9)
O1—Cd—Ni—N1	−16.5 (5)	Ni—N2—C11—C12	−61.4 (10)
O3^i^—Cd—Ni—N1	−94.3 (4)	C13—C12—C11—N2	−115.3 (11)
O3—Cd—Ni—N1	85.7 (4)	C17—C12—C11—N2	66.0 (13)
O2—Cd—Ni—N2	15.8 (4)	Ni—O1—C1—C2	−134.8 (10)
O2^i^—Cd—Ni—N2	−164.2 (4)	Cd—O1—C1—C2	9.5 (16)
O1^i^—Cd—Ni—N2	−6.8 (5)	Ni—O1—C1—C6	46.8 (12)
O1—Cd—Ni—N2	173.2 (5)	Cd—O1—C1—C6	−168.9 (8)
O3^i^—Cd—Ni—N2	95.4 (4)	Ni—O2—C17—C16	135.0 (9)
O3—Cd—Ni—N2	−84.6 (4)	Cd—O2—C17—C16	−7.2 (16)
O2—Cd—Ni—O5	−76.2 (4)	Ni—O2—C17—C12	−47.0 (12)
O2^i^—Cd—Ni—O5	103.8 (4)	Cd—O2—C17—C12	170.7 (8)
O1^i^—Cd—Ni—O5	−98.8 (4)	C13—C12—C17—O2	177.2 (10)
O1—Cd—Ni—O5	81.2 (4)	C11—C12—C17—O2	−4.2 (16)
O3^i^—Cd—Ni—O5	3.4 (3)	C13—C12—C17—C16	−4.8 (16)
O3—Cd—Ni—O5	−176.6 (3)	C11—C12—C17—C16	173.9 (10)
O1—Ni—O5—C20	42.4 (9)	O2—C17—C16—C15	−177.1 (11)
O2—Ni—O5—C20	−41.0 (8)	C12—C17—C16—C15	4.9 (17)
N1—Ni—O5—C20	134.4 (9)	C7—N1—C8—C9	174.4 (10)
N2—Ni—O5—C20	−133.1 (9)	Ni—N1—C8—C9	−60.2 (11)
Cd—Ni—O5—C20	0.2 (9)	O1—C1—C6—C5	−175.0 (11)
O1—Ni—O2—C17	−139.4 (8)	C2—C1—C6—C5	6.5 (17)
O4—Ni—O2—C17	126.8 (7)	O1—C1—C6—C7	3.1 (16)
N2—Ni—O2—C17	36.8 (8)	C2—C1—C6—C7	−175.3 (11)
O5—Ni—O2—C17	−46.5 (7)	Ni—O5—C20—N3	150.1 (10)
Cd—Ni—O2—C17	−154.6 (9)	C22—N3—C20—O5	−3(2)
O1—Ni—O2—Cd	15.2 (3)	C21—N3—C20—O5	−176.7 (13)
O4—Ni—O2—Cd	−78.6 (3)	C11—N2—C10—C9	−175.7 (9)
N2—Ni—O2—Cd	−168.6 (3)	Ni—N2—C10—C9	58.4 (11)
O5—Ni—O2—Cd	108.1 (3)	C1—C6—C5—C4	−4(2)
O1^i^—Cd—O2—C17	−46.8 (10)	C7—C6—C5—C4	177.6 (14)
O1—Cd—O2—C17	133.2 (10)	O1—C1—C2—C3	176.3 (11)
O3^i^—Cd—O2—C17	39.1 (10)	C6—C1—C2—C3	−5.3 (18)
O3—Cd—O2—C17	−140.9 (10)	N2—C10—C9—C8	−69.7 (13)
Ni—Cd—O2—C17	147.7 (11)	N1—C8—C9—C10	71.7 (13)
Ni^i^—Cd—O2—C17	−32.3 (11)	C17—C16—C15—C14	−2.2 (19)
O1^i^—Cd—O2—Ni	165.5 (3)	C17—C12—C13—C14	2.1 (17)
O1—Cd—O2—Ni	−14.5 (3)	C11—C12—C13—C14	−176.6 (11)
O3^i^—Cd—O2—Ni	−108.7 (3)	O1—Ni—O4—C18	−44.8 (8)
O3—Cd—O2—Ni	71.3 (3)	O2—Ni—O4—C18	38.6 (8)
Ni^i^—Cd—O2—Ni	180	N1—Ni—O4—C18	−137.2 (9)
O4—Ni—O1—C1	−130.3 (8)	N2—Ni—O4—C18	130.6 (9)
O2—Ni—O1—C1	140.3 (8)	Cd—Ni—O4—C18	−3.5 (8)
N1—Ni—O1—C1	−36.3 (8)	O2—Cd—O3—C18	−30.5 (9)
O5—Ni—O1—C1	51.1 (8)	O2^i^—Cd—O3—C18	149.5 (9)
Cd—Ni—O1—C1	155.5 (9)	O1^i^—Cd—O3—C18	−131.6 (9)
O4—Ni—O1—Cd	74.2 (3)	O1—Cd—O3—C18	48.4 (9)
O2—Ni—O1—Cd	−15.2 (3)	Ni—Cd—O3—C18	9.5 (8)
N1—Ni—O1—Cd	168.1 (3)	Ni^i^—Cd—O3—C18	−170.5 (8)
O5—Ni—O1—Cd	−104.4 (3)	Cd—O3—C18—O4	−17.1 (17)
O2—Cd—O1—C1	−134.8 (9)	Cd—O3—C18—C19	160.6 (7)
O2^i^—Cd—O1—C1	45.2 (9)	Ni—O4—C18—O3	12.9 (16)
O3^i^—Cd—O1—C1	−39.7 (9)	Ni—O4—C18—C19	−164.9 (7)
O3—Cd—O1—C1	140.3 (9)	C16—C15—C14—C13	−1(2)
Ni—Cd—O1—C1	−149.6 (10)	C12—C13—C14—C15	0.8 (18)
Ni^i^—Cd—O1—C1	30.4 (10)	C1—C2—C3—C4	2(2)
O2—Cd—O1—Ni	14.8 (3)	C6—C5—C4—C3	1(2)
O2^i^—Cd—O1—Ni	−165.2 (3)	C2—C3—C4—C5	1(2)
O3^i^—Cd—O1—Ni	109.9 (3)	C8—N1—C7—C6	−170.3 (11)
O3—Cd—O1—Ni	−70.1 (3)	Ni—N1—C7—C6	61.8 (12)
Ni^i^—Cd—O1—Ni	180	C5—C6—C7—N1	113.1 (14)
O4—Ni—N2—C10	52.3 (8)	C1—C6—C7—N1	−64.9 (15)
O2—Ni—N2—C10	142.1 (8)		

Symmetry codes: (i) −*x*, −*y*, −*z*.

### Hydrogen-bond geometry (Å, °)

**Table:**

*D*—H···*A*	*D*—H	H···*A*	*D*···*A*	*D*—H···*A*
C2—H2···O3^ii^	0.93	2.56	3.308 (16)	138
C20—H20···O3^ii^	0.93	2.59	3.409 (14)	147

Symmetry codes: (ii) −*x*, −*y*, −*z*.
